# First laboratory-confirmed fatal dengue virus infection and its implications for national arboviral surveillance in Liberia

**DOI:** 10.1016/j.ijregi.2026.101014

**Published:** 2026-09-11

**Authors:** Bode Ireti Shobayo, Chukwuma David Umeokonkwo, Kalilu Samuka Donzo, Methodius George, Emmanuel Dwalu, Ralph Jetoh, Fahn Taweh, Julius Gilayeneh, Sia Wata Camanor, Stephen B. Kennedy

**Affiliations:** 1 National Public Health Institute of Liberia (NPHIL), Monrovia, Liberia; 2 Karolinska Institutet, Solna, Stockholm, Sweden; 3 African Field Epidemiology Network (AFENET), Kampala, Uganda; 4 Ministry of Health (MoH), Republic of Liberia, Monrovia, Liberia; 5 UL-PIRE Africa Center, An Infectious Disease Research Center, University of Liberia, Monrovia, Liberia

**Keywords:** Dengue virus, Fatal dengue infection, Arboviral surveillance, Maryland County, Liberia

## Abstract

**Introduction:**

Dengue virus (DENV) is an emerging public health threat in Africa, where its impact is underestimated due to nonspecific clinical manifestations and restricted diagnostic capabilities. In Liberia, laboratory-confirmed cases of DENV were initially recorded in an urban febrile surveillance study (2018-2020), yet severe or fatal outcomes had not been previously reported. This paper reports the first laboratory-confirmed fatal dengue case and its implications for national surveillance and policy in Liberia.

**Case report:**

On February 2, 2026, a 38-year-old Liberian man arrived at J. J. Dossen Memorial Hospital in Maryland County, located in the southern region of Liberia, with a 2-day history of high fever, severe headache, myalgia, weakness, and abdominal discomfort. He had no recent history of travel. The first laboratory investigation using a rapid test and microscopy ruled out malaria. Molecular detection performed at the National Public Health Reference Laboratory was negative for Lassa, Ebola, and Marburg. Despite management with supportive care, his condition deteriorated clinically upon readmission, and the patient died on February 6, 2026. Serological testing performed at MDS-Lancet Laboratories, Ghana detected DENV immunoglobulin G antibodies and yielded a positive nonstructural protein 1 antigen result, prompting further investigations. Consequently, a blood sample was referred to the Institut Pasteur de Dakar, Senegal, where confirmatory testing was conducted to include differential reverse transcription polymerase chain reaction for a diverse array of arboviruses and viral hemorrhagic fevers and differential virus-specific immunoglobulin M (IgM) enzyme-linked immunosorbent assay. The sample tested positive for DENV ribonucleic acid by reverse transcription polymerase chain reaction, negative for other viruses, and negative for dengue-specific IgM. The absence of detectable IgM may reflect specimen collection during an early stage of infection before seroconversion. However, IgM negativity alone cannot distinguish between primary and secondary infection.

**Conclusion:**

This is the first molecular confirmation of fatal dengue in Liberia. It highlights the potential for severe outcomes and suggests locally acquired transmission in southeastern Liberia, a region bordering Côte d'Ivoire. There is a critical need for swift implementation of integrated arboviral surveillance in both urban and rural settings, better diagnostic capabilities, and possibly a nationwide seroepidemiological study to estimate the actual burden of dengue in the country.

## Highlights

• First laboratory-confirmed fatal dengue virus infection reported in Liberia.
• Dengue infection was confirmed after malaria and other infections were ruled out.
• Severe dengue caused bleeding, shock, and failure of multiple organs.
• No travel history suggests local dengue transmission in rural Liberia.
• Findings support stronger mosquito-borne disease surveillance and testing.

## Introduction

Dengue fever, caused by four antigenically diverse serotypes of dengue virus (DENV; DENV-1, -2, -3, and -4) and largely transmitted by *Aedes aegypti* mosquitoes, is the most rapidly expanding mosquito-borne viral illness worldwide [1,2]. Dengue infections account for about 390 million cases each year, of which approximately 96 million present with clinical symptoms [3]. Historically, the Americas and Southeast Asia have recorded the highest incidence of dengue. However, it is becoming an increasing public health threat in Africa due to increasing urbanization and human mobility, climate change, and the widespread distribution of competent mosquito vectors across the continent [4,5].

Africa is considered endemic for all four DENV serotypes (DENV-1, DENV-2, DENV-3, and DENV-4), with DENV-2 implicated in most of the recent outbreaks across the continent. The primary vector, *Aedes aegypti*, has been documented in 47 of the 55 African Union member states; however, dengue cases have been reported in only 34 of these countries. Compared with 2019, the burden of dengue in Africa increased dramatically, rising ninefold by December 2023 to more than 270,000 reported cases and 753 deaths across 18 African Union member states [6]. Because dengue shares many clinical features with endemic febrile illnesses, especially malaria, a considerable proportion of cases are frequently misdiagnosed and treated as malaria. This diagnostic overlap not only obscures the true epidemiological burden of dengue in endemic settings but also promotes unnecessary antimalarial use, potentially contributing to the development and propagation of antimalarial drug resistance [7].

Dengue vaccine development has advanced substantially in recent years, but major gaps remain in vaccine licensure and accessibility across Africa [8]. Dengvaxia (CYD-TDV), licensed in 2015 as the first dengue vaccine, demonstrated moderate efficacy against symptomatic dengue but was later found to increase the risk of severe disease among seronegative individuals, leading to highly restrictive indications that require laboratory-confirmed prior dengue infection before vaccination [9,10]. In contrast, Qdenga (TAK-003), licensed since 2022 in at least 39 countries, has shown approximately 80% efficacy in preventing virologically confirmed dengue and does not require mandatory prevaccination serostatus screening, although recent long-term studies have highlighted variable protection across serotypes and ongoing concerns regarding potential risks among seronegative recipients [11,12]. A third dengue vaccine, Butantan-DV, was recently approved in Brazil following phase III trial results showing 65% efficacy against symptomatic dengue and 80.5% protection against severe disease, although evidence remains limited to DENV-1 and DENV-2 [13]. Despite its promise as the first licensed single-dose dengue vaccine, rollout was temporarily suspended in June 2026 pending investigation of two deaths and 42 severe adverse events reported among approximately 500,000 vaccine recipients [14]. Despite increasing dengue transmission and likely underreporting, no dengue vaccine is currently licensed in sub-Saharan Africa. This highlights the need for stronger surveillance and evidence to inform vaccine licensure and introduction across the continent.

Dengue outbreaks or cases have been documented in countries neighboring Liberia, such as Guinea, Sierra Leone, and Côte d'Ivoire [[15], [16], [17]]. In 2023-2024, Côte d'Ivoire recorded hundreds of confirmed DENV cases [8,18]. The region is considered at high risk for transmission, wherein 10 Economic Community of West African States nations have documented outbreaks [18]. Malaria is endemic in Liberia, which has a history of recurrent outbreaks of infectious diseases, including vector-borne diseases such as yellow fever and rodent-borne diseases such as Lassa fever [[19], [20], [21]]. However, documented evidence of DENV circulation has only recently begun to emerge [18,22,23]. Between December 2018 and March 2020, an acute febrile illness surveillance study was conducted in two urban health facilities in Monrovia, Montserrado County, to evaluate the common causes of febrile illnesses with TaqMan Array Cards. This study reported the presence of the first laboratory-confirmed cases of DENV in 2% of the study population [24]. This study was, however, restricted to the capital city, whereas the epidemiological burden was uncharacterized in other regions of the country. Critically, the clinical spectrum of dengue across Liberia, including severe manifestations and fatalities, remains undescribed.

The fatal case described in this paper, which occurred in a physician in rural southeastern Liberia, highlights a sentinel event. Additionally, it confirmed the presence of fatal dengue fever infection in a resource-limited setting with unprepared clinical and laboratory diagnostic infrastructure and an increased likelihood of increased mortality. This paper aimed to provide a comprehensive analysis of this unprecedented case and to explore its implications for the enhancement of Liberia's national arboviral surveillance, diagnostic capacity, clinical management guidelines, and the justification for a potential countrywide seroepidemiological study.

## Case presentation

A 38-year-old Liberian male physician presented to the outpatient department of J. J. Dossen Memorial Hospital in Harper, Maryland County, southeastern Liberia, on February 2, 2026, with a history of acute febrile illness characterized by high-grade fever, severe headache, generalized myalgia, profound weakness, and abdominal discomfort. He was previously well with a reported history of sickle cell trait and chronic peptic ulcer disease managed with long-term omeprazole therapy. He had no history of hypertension, diabetes mellitus, or retroviral disease. On examination, he appeared ill but hemodynamically stable, with a blood pressure of 120/82 mmHg, pulse rate of 99 beats/min, respiratory rate of 20 breaths/min, temperature of 37.5°C, and oxygen saturation of 99% on room air. Physical examination was largely unremarkable except for mild mucosal pallor. Initial laboratory investigations revealed a white blood cell count of 7.1 × 10⁹/l, hemoglobin of 13.2 g/dl, and marked thrombocytopenia with a platelet count of 19 × 10⁹/l. Renal function and electrolytes were within normal limits, and blood cultures showed no growth. He also reported no recent travel history outside of Liberia and had no known exposure to a patient with confirmed hemorrhagic fever.

Initial clinical evaluation considered common causes of acute febrile illnesses in the region. Laboratory investigations performed at the National Public Health Reference Laboratory ruled out several major endemic and epidemic-prone infections, including Lassa, Ebola, and Marburg, through molecular testing. Malaria testing, including rapid diagnostic testing and confirmatory microscopy, was negative. He was managed empirically with intravenous artesunate and ceftriaxone along with supportive care, with transient clinical improvement, and was initially discharged. Within 24 h after discharge, he was readmitted with hematemesis, passage of melenic stools, severe epigastric pain, progressive abdominal distension, generalized weakness, dizziness, palpitations, and shortness of breath. On examination, he was markedly pale with tachycardia (149 beats/min), a distended abdomen with evidence of ascites, generalized abdominal tenderness, and reduced urine output. Laboratory evaluation at this stage demonstrated marked leukocytosis (30.4 × 10⁹/l), worsening anemia (hemoglobin 10.8 g/dl), and severe thrombocytopenia (platelets 7 × 10⁹/l). Serum lactate was significantly elevated at 6.65 mmol/l, consistent with severe lactic acidosis. Renal function had deteriorated (creatinine 1.9 mg/dl), and liver enzymes were elevated. Abdominal ultrasound revealed free intraperitoneal fluid, while plain radiography showed no evidence of perforation. He was managed for severe sepsis with suspected intra-abdominal pathology, possible viral hemorrhagic fever, and evolving multiorgan dysfunction. Treatment included intravenous meropenem, blood transfusions, proton pump inhibitor therapy, oxygen supplementation, and intensive monitoring. Arterial blood gas analysis confirmed severe metabolic acidosis.

Due to worsening abdominal signs, an exploratory laparotomy was performed. Intraoperative findings included approximately 1100 mL of serous peritoneal fluid, with no evidence of perforation, pus, a bleeding source, obstruction, or pancreatitis. The bowel loops were dilated, there was a marked bleeding tendency, and the spleen appeared enlarged and engorged. Postoperatively, the patient’s condition deteriorated rapidly with refractory shock and progressive multiorgan failure. Further laboratory investigations demonstrated extreme leukocytosis (51.6 × 10⁹/l) with neutrophilia and relative lymphopenia, persistent thrombocytopenia, and markedly elevated liver enzymes, including alanine aminotransferase of 5160 IU/l. Peripheral blood smear findings suggested a leukemoid reaction versus acute hematologic malignancy. Despite aggressive resuscitative efforts, he developed cardiac arrest within 8-9 h following surgery, could not be revived, and died on February 6, 2026.

Given the unexplained clinical course, additional laboratory investigations were pursued. Serological testing performed at MDS-Lancet Laboratories, Ghana detected immunoglobulin G (IgG) antibodies and yielded a positive nonstructural protein 1 (NS1) antigen result. The specimen was collected in Liberia and referred for testing at MDS-Lancet Laboratories in Ghana. However, detailed information regarding the NS1 antigen assay platform, manufacturer, testing methodology, and specimen collection date was not available to the authors because testing was performed externally. The IgG result was interpreted cautiously because flavivirus serological assays may demonstrate cross-reactivity with antibodies generated against other flaviviruses. These findings prompted referral of a specimen for confirmatory testing at the Institut Pasteur de Dakar, Senegal.

On March 3, 2026, a blood specimen collected in Liberia was received at the Institut Pasteur de Dakar in Senegal, a World Health Organization Collaborating Centre for arboviruses and viral hemorrhagic fevers. The specimen (IPD number 504802; laboratory number LIB-BVD26-025) arrived in good condition and underwent a comprehensive diagnostic evaluation. Serological testing was performed using a differential enzyme-linked immunosorbent assay to detect virus-specific immunoglobulin M (IgM) against a panel of arboviruses and hemorrhagic fever viruses. In parallel, differential real-time reverse transcription polymerase chain reaction (RT-PCR) assays were performed for arboviruses and viral hemorrhagic fever viruses. The enzyme-linked immunosorbent assay did not detect dengue-specific IgM antibodies. However, because complete specimen collection metadata were unavailable and symptom onset preceded testing by several days, the significance of the negative IgM result is uncertain. The available laboratory findings do not permit distinction between primary and secondary dengue infection. However, RT-PCR testing revealed a positive result for DENV ribonucleic acid (RNA), confirming the presence of DENV infection. The laboratory report did not include RT-PCR cycle threshold values, assay platform details, or DENV serotype information. PCR assays targeting other viral pathogens, including yellow fever, chikungunya, West Nile, Zika, Rift Valley fever, Crimean-Congo hemorrhagic fever, Ebola, Marburg, and Lassa viruses, were all negative (Table 1).

**Table 1:** Timeline of clinical presentation, diagnostic investigations, and laboratory confirmation of fatal dengue infection in Liberia.

Date	Event
January 31, 2026	Symptom onset
February 2, 2026	First presentation: malaria RDT/microscopy negative
February 6, 2026	Date of sample collection
February 7, 2026	Molecular testing for Lassa, Ebola, and Marburg negative (NPHRL)
February 9, 2026	NS1 positive (MDS-Lancet Laboratories in Ghana)
February 9, 2026	IgG positive (MDS-Lancet Laboratories in Ghana)
February 9, 2026	IgM negative (MDS-Lancet Laboratories in Ghana)
March 4, 2026	RT-PCR dengue positive (Institut Pasteur de Dakar)
	RT-PCR for other viral pathogens negative (Institut Pasteur de Dakar)
	IgM negative (Institut Pasteur de Dakar)

IgG, immunoglobulin G; IgM, immunoglobulin M; NPHRL, National Public Health Reference Laboratory; NS1, nonstructural protein 1; RDT, rapid diagnostic test; RT-PCR, reverse transcription polymerase chain reaction.

Collectively, the results were interpreted as consistent with a recent DENV infection that occurred during the viremic phase, thereby excluding other major arboviral and viral hemorrhagic fever pathogens as etiologic agents. The laboratory confirmation of DENV RNA in this fatal case provides important evidence of dengue-associated mortality in Liberia and highlights the diagnostic complexity of acute febrile illness in settings where multiple endemic pathogens present with overlapping clinical manifestations.

## Discussion

This represents, to our knowledge, the first laboratory-confirmed fatal DENV infection reported in Liberia. Although DENV circulation has previously been documented among patients with acute febrile illness in Monrovia, severe disease and dengue-associated mortality have not previously been described in the country. The case therefore provides important evidence that dengue can cause life-threatening illness in Liberia and highlights the need for greater clinical awareness and improved diagnostic capacity.

The clinical presentation underscores the diagnostic challenges associated with acute febrile illness in West Africa. The patient initially presented with nonspecific symptoms, including fever, severe headache, myalgia, weakness, and abdominal discomfort, which overlap substantially with malaria, Lassa fever, and other endemic infections [25,26]. Malaria testing was negative, and subsequent molecular testing excluded Ebola, Marburg, and Lassa fever. However, dengue was not initially suspected because dengue diagnostics are not routinely available, and arboviral infections are not commonly prioritized in the differential diagnosis of febrile illness in Liberia. This diagnostic uncertainty illustrates how dengue infections may remain unrecognized until severe complications develop.

The laboratory findings provide important insights into the progression of disease in this patient. Detection of DENV RNA by RT-PCR provided definitive evidence of acute dengue infection, while the positive NS1 antigen result further supported recent infection. The negative dengue IgM result is compatible with specimen collection during the early viremic phase before antibody seroconversion, when molecular and antigen-based assays are generally more sensitive than serological testing [27]. DENV IgG antibodies were also detected; however, because flavivirus serological assays may exhibit cross-reactivity with antibodies to other flaviviruses, this finding should be interpreted cautiously. Consequently, the available laboratory data are insufficient to classify the infection as either primary or secondary dengue infection. Furthermore, detailed laboratory metadata, including RT-PCR cycle threshold values and dengue serotype information, were not available from the referral laboratory.

Several clinical and laboratory findings were consistent with severe dengue. The patient developed profound thrombocytopenia, with platelet counts declining from 19 × 10⁹/l to 7 × 10⁹/l, accompanied by hematemesis, melena, ascites, shock, hepatic injury, renal impairment, metabolic acidosis, and progressive multiorgan dysfunction. These findings are compatible with severe dengue characterized by plasma leakage, hemorrhagic manifestations, and organ involvement [28]. The severity of thrombocytopenia likely contributed to the patient's hemorrhagic manifestations. The rapid clinical deterioration despite supportive management demonstrates the potential severity of dengue infection and the challenges of managing patients with severe disease in resource-limited settings, where diagnosis may be delayed.

The patient also had a reported history of sickle cell trait and chronic peptic ulcer disease that may have influenced clinical assessment and management. However, confirmatory hemoglobin electrophoresis results were unavailable, and the diagnosis of sickle cell trait could not be independently verified. Sickle cell trait is generally considered clinically benign and is unlikely to have been a major determinant of outcome [29]. In contrast, pre-existing peptic ulcer disease may have complicated the clinical assessment of gastrointestinal bleeding, particularly following the development of hematemesis and melena. Nevertheless, the extent to which this underlying condition contributed to the patient's deterioration remains uncertain, given the concurrent presence of severe thrombocytopenia, hemorrhagic manifestations, shock, and multiorgan dysfunction attributable to acute DENV infection.

An unusual feature of this case was the development of marked leukocytosis (51.6 × 10⁹/l), whereas leukopenia is more commonly reported in dengue infection. Although uncommon, severe dengue complicated by shock, hemorrhage, profound systemic inflammation, or leukemoid reactions has been associated with marked leukocytosis [30]. In this patient, the progressive shock state, severe bleeding manifestations, multiorgan dysfunction, and postoperative physiological stress may have contributed to the extreme leukocyte response. However, alternative diagnoses such as leptospirosis or occult bacterial infection could not be completely excluded [31] because specific testing was unavailable.

The epidemiological context of this case suggests locally acquired dengue infection in Maryland County. The patient resided and worked in the county and reported no recent travel history outside Liberia, which renders importation unlikely. Maryland County lies along an active cross-border corridor with Côte d'Ivoire and possesses environmental conditions favorable for *Aedes* mosquito breeding, including high rainfall, tropical vegetation, and expanding semiurban settlements. Unlike previous reports of DENV circulation from Monrovia [24], this fatal case occurred in rural southeastern Liberia, suggesting that transmission may be more geographically widespread than currently recognized.

This case has important public health implications. First, it demonstrates that dengue should be considered in the differential diagnosis of severe acute febrile illness, especially when malaria and viral hemorrhagic fevers have been excluded. Second, it highlights the need to strengthen access to dengue diagnostics, including antigen and molecular testing, to facilitate early detection and appropriate clinical management. Finally, it underscores the importance of integrating arboviral diseases into national surveillance systems and clinical management algorithms to improve recognition of severe dengue and reduce the risk of future fatalities.

Limitations of this report include the absence of laboratory data from the referral laboratories, such as the specific NS1 assay, RT-PCR cycle threshold values, and dengue serotyping results. Furthermore, hemoglobin electrophoresis findings were not available to verify the reported history of sickle cell trait. Specific testing for leptospirosis was also unavailable, and therefore, this alternative diagnosis could not be conclusively excluded.

## Conclusion

This fatal dengue case in Liberia illustrates the virus’s capacity to induce severe disease and mortality, and underscores its importance as a significant public health issue. The ambiguous febrile symptoms and rapid deterioration underscore the diagnostic challenges presented by overlapping endemic diseases and constrained laboratory capabilities. The confirmation of locally acquired transmission in Maryland County reveals that the transmission of dengue fever extends beyond Monrovia, implying a wider, unreported circulation. These findings underscore the imperative to strengthen surveillance systems, expand diagnostic capacity, enhance preparedness and surveillance at Points of Entry (PoE), and train healthcare professionals in the application of clinical algorithms for the timely identification and management of patients presenting with febrile illnesses. These findings highlight the need to strengthen arboviral surveillance, expand access to dengue diagnostics, and improve clinician awareness of dengue as a cause of severe acute febrile illness. Early recognition and appropriate supportive management may help reduce dengue-associated morbidity and mortality in Liberia.

## Declarations of competing interest

The authors have no competing interests to declare.

## Funding

The authors received no funding for this research.

## Ethical approval

This report represents a retrospective single-patient case report based on data collected during routine clinical care and public health surveillance activities. It did not involve research interventions, prospective participant recruitment, or experimental procedures. Accordingly, formal ethics review was not required under applicable institutional and national guidelines.

This report describes a single retrospective case identified through routine clinical care and public health surveillance activities and did not involve research interventions, prospective participant recruitment, or experimental procedures. Accordingly, formal ethics committee review was not required under applicable institutional and national guidelines.

**Informed consent and patient details**: The patient was deceased at the time of manuscript preparation. Consent for publication was not obtained from a legal representative or next of kin. This case report was based on anonymized clinical and public health surveillance data collected as part of routine patient management and public health investigations. No direct identifiers are is reported. The authors considered the risk of patient identification to be minimal; however, certain clinical and epidemiological details were retained because they were necessary for scientific interpretation of the case.

**Studies in human:** This study was performed in compliance with relevant laws, regulatory frameworks and guidelines where the research took place.

Ethics committee approval was not required under relevant laws and institutional guidelines. This report is a retrospective single-patient case report based on information collected during routine clinical care and public health surveillance activities. No research intervention, experimental procedure, or predefined study hypothesis was involved. Consequently, formal ethical approval was not required under applicable institutional and national regulations. Direct personal identifiers, including the patient's name, address, and other unique personal identifiers, were removed before analysis and reporting. Certain demographic, clinical, laboratory, and epidemiological details were retained because they were considered essential for interpretation of this case report.

**Case reports**: This research follows the CAse REports (CARE) guidelines and the CARE checklist.

## CRediT authorship contribution statement

**Bode Ireti Shobayo:** Conceptualization, Investigation, Writing – original draft. **Chukwuma David Umeokonkwo:** Methodology, Writing – review & editing. **Kalilu Samuka Donzo:** Data curation, Investigation, Writing – review & editing. **Methodius George:** Data curation, Investigation, Methodology, Writing – review & editing. **Emmanuel Dwalu:** Investigation, Writing – review & editing. **Ralph Jetoh:** Writing – review & editing. **Fahn Taweh:** Investigation, Writing – review & editing. **Julius Gilayeneh:** Supervision, Writing – review & editing. **Sia Wata Camanor:** Supervision, Writing – review & editing. **Stephen B. Kennedy:** Supervision, Writing – review & editing.
